# Engineering-geological study of relationships between soil and rock workability, type and volume of excavated materials, and earthwork costs (six case studies)

**DOI:** 10.1038/s41598-023-31859-3

**Published:** 2023-04-02

**Authors:** Marian Marschalko, Dariusz Popielarczyk, Simona Matuskova, Dominik Niemiec, David Neuman, Veronika Glacova

**Affiliations:** 1 Department of Geological Engineering, Faculty of Mining and Geology, VŠB-Technical University of Ostrava, 708 33 Ostrava, Czech Republic; 2 Department of Geodesy, Faculty of Geoengineering, University of Warmia and Mazury in Olsztyn, Oczapowskiego 2, 10-719 Olsztyn, Poland; 3 Department of Environmental Engineering, Faculty of Mining and Geology, VŠB-Technical University of Ostrava, 708 33 Ostrava, Czech Republic

**Keywords:** Environmental economics, Urban ecology, Ecology, Environmental sciences, Natural hazards, Solid Earth sciences

## Abstract

The engineering-geological study deals with the study of significance and relationship of soil and rock workability (factor representing the engineering-geological structure of rock massif) and the remaining earthwork parameters influencing the cost of construction work, such as excavation type and its technology, and excavated cubic volume. The comparative tool was the cost of earthwork as it reflects the real value of the given parameters during the implementation of earthwork. Soil and rock workability is the most important parameter of rock massif engineering-geological structure during any earthwork. The investor pays the contractor for earthwork based on workability classes which have their accounting value expressed as a volume unit of earthwork per particular project. The research results arise from a comparison of 6 sewer system construction project case studies in the north-east of the Czech Republic. The research shows that the most important factor during the implementation of earthwork is the specific engineering-geological structure (52%), which reflects in the parameter of soil and rock workability classes, using which all earthwork is priced. The second most important factor (33%) is the type of excavation and its technology. The least important is the excavated cubic volume (15%), which means the overall cubic volume of earthwork. The results were obtained within three evaluation approaches, where the comparison unit was one cubic meter of excavated volume during earthwork.

## Introduction

**Figure 1:**
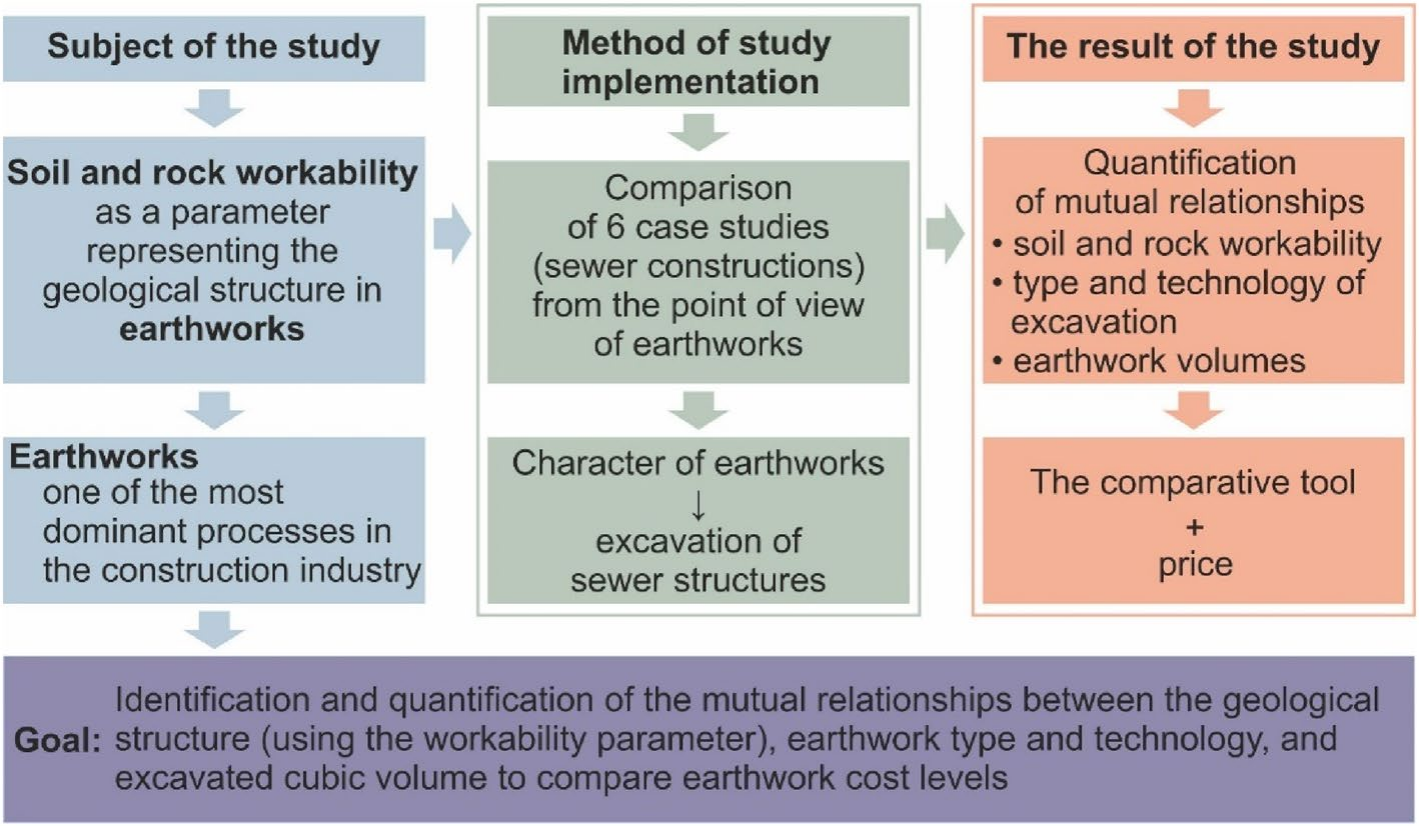
Simplified scheme of the research goals.

The motivation and subject of the engineering-geological study (Fig. 1) is to identify the significance of the character of geological structure (represented by soil and rock workability) on earthwork implementation^1–4^. To do that, it is also important to verify the significance of other parameters (type, technology and volume of excavation).

All these parameters (factors) participate on the costs of earthwork, and thus the comparison unit will be the cost of excavated volume (m^3^). Such information is important for investors and contractors (also for engineering geologists or geotechnicians) carrying out earthwork. Knowing the costs of each parameter means that due attention is paid during planning to predict the course of the works and to avoid possible pitfalls. The study also has scientific and didactic goals as it may help prepare researchers, teachers and students to approach such matters. Earthwork, which is the subject of this study, represents an important component of each construction project. It also makes part of all engineering-geological investigations^5–7^. Moreover, in some construction projects, earthwork is the dominant work, e.g., in sewer system or road construction. To carry out this study, we chose sewer system construction projects and compared 6 different sewer system construction projects as case studies from the north-east of the Czech Republic (Fig. 1). Sewer systems have been discussed by Xu et al.^8^ and Kang et al.^9^, but in the context of geohazards or earthquakes.

The study results in quantified relationships of soil and rock workability, and the type, technology and volume of excavation using a comparative tool of earthwork costs (price for excavating 1 m^3^).

Soil and rock workability^10–14^ is a property characterized by the amount of work needed to break and load soil or rocks during earthwork related to a volume unit. It corresponds to the resistance the soil or rock exerts towards loosening (identical to the breaking characteristic of rocks), takes into account clinging (stickiness) of rocks onto tools, loosening and difficulty in loading onto vehicles. It is expressed by 7 workability classes^15,16^. The most similar property is the breaking characteristic of rocks^17–21^, but it does not take into account work needed to load the worked material onto a vehicle. In earthwork, environmental aspects must also be considered^22,23^.

**Figure 2:**
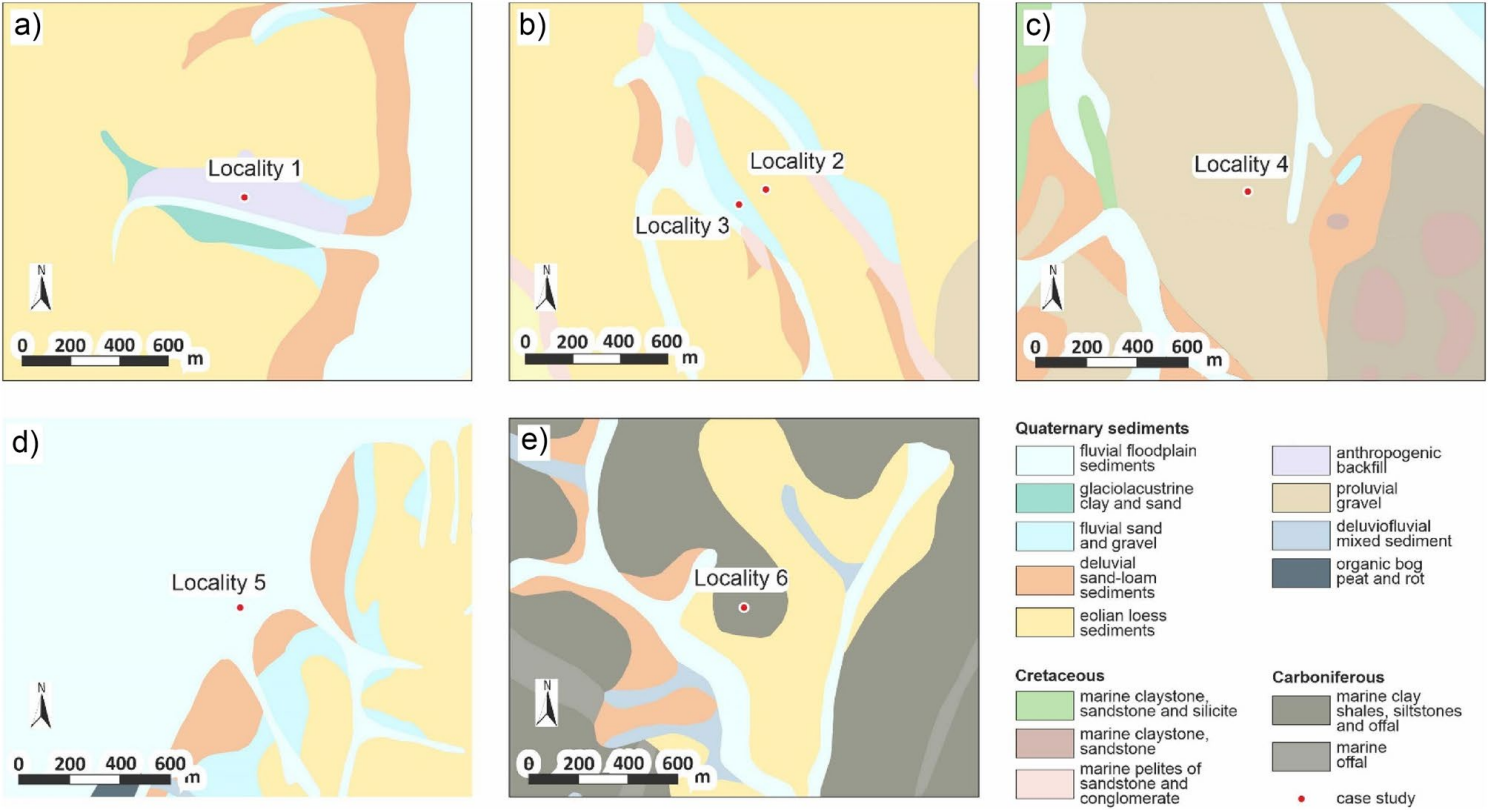
Location of case studies in the geological map (**a**) locality 1, (**b**) localities 2 and 3, (**c**) locality 4, (**d**) locality 5, (**e**) locality 6. Schematic figures made by the authors using CorelDRAW Graphic Suite 2019 software www.coreldraw.com.

From the geological point of view, the six localities (case studies, Fig. 2) are found in the anthropogenic backfills (locality 1), eolian loess sediments (locality 2), fluvial sand and gravel (locality 3), proluvial gravel (locality 4), fluvial floodplain clayey, sandy and gravel-sandy sediments (locality 5), and marine eluvial pelites and psamites (locality 6). All excavations in the stated localities were executed in Quaternary sediments, only in the sixth locality the preQuaternary engineering-geological structure formed by Carboniferous marine pelites was also excavated.

As for geomorphology, localities 1 and 5 are in the Outer Carpathian Depressions; looking at more detailed geomorphology, locality 1 is in Havířov Plateau of the Ostrava Basin and locality 5 in the Odra floodplain of the Moravian Gate. Localities 2, 3 and 4 are found in the Western Carpathians and Lower Beskydy Uplands. While localities 2 and 3 are in the Frýdek Upland, locality 4 falls into Radhošť Foothills. Locality 6 is situated in the Děhylov Upland of the Lower Jeseník belonging to the Krkonoše-Jeseník Subprovince.

**Figure 3:**
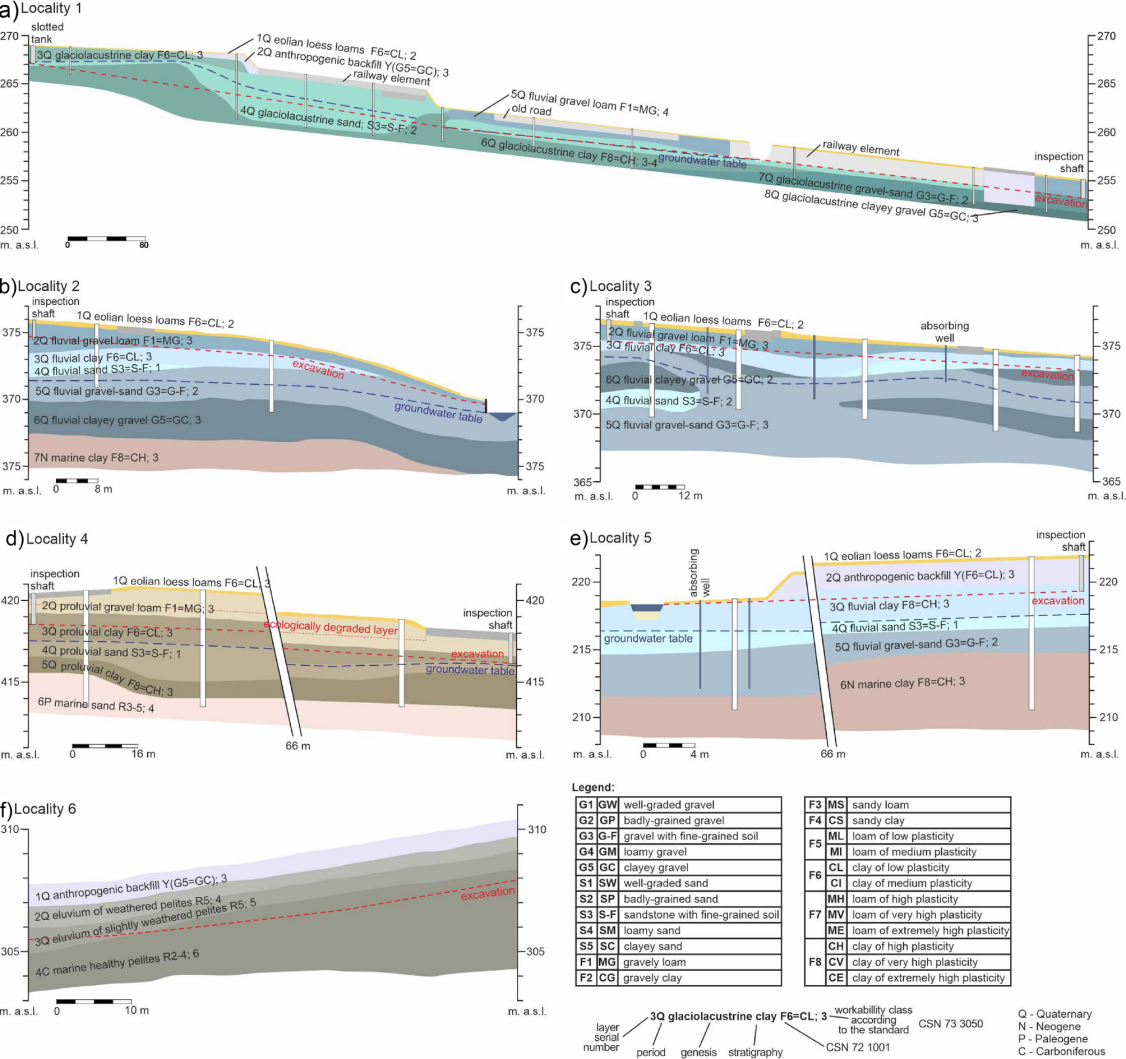
Engineering-geological sections of the case studies (**a**) locality 1, (**b**) locality 2, (**c**) locality 3, (**d**) locality 4, (**e**) locality 5, (**f**) locality 6. Schematic figures made by the authors using CorelDRAW Graphic Suite 2019 software www.coreldraw.com.

From the engineering-geological point of view, the subsoil layers of the sewer system in the *locality of the first case study* must be divided into excavated and non-excavated layers (Fig. 3). The engineering structure was 1.1 km long. The excavated layers were (stated from the ground surface down; left to right): eolian loess loams (soil type F6 = CL Fig. 3—legend; workability class—t.t. 2), anthropogenic backfill (Y with the character of G5 = GC; t.t. 3), glaciolacustrine clay (F6 = CL; t.t. 3), fluvial gravel loam (F1 = MG; t.t. 4) and glaciolacustrine sand (S3 = S-F; t.t. 2) and glaciolacustrine clay (F8 = CH; t.t. 3). The last two layers were found both in the excavated and non-excavated part of the subsoil (see the engineering-geological section). The non-excavated part constitutes of glaciolacustrine gravel-sand (G3 = G-F; t.t. 2) and glaciolacustrine clayey gravel (G5 = GC; t.t. 3).

*In the locality of the second case study* (Fig. 3b) the excavated layers were eolian loess loams (soil type F6 = CL; workability class—t.t. 2), fluvial gravel loam (F1 = MG; t.t. 3), fluvial clay (F6 = CL; t.t. 3) and fluvial gravel-sand (G3 = G-F; t.t. 2). The last three layers were both in the excavated and non-excavated part. The remaining non-excavated subsoil layers were fluvial sand (S3 = S-F; t.t. 1), fluvial clayey gravel (G5 = GC; t.t. 3) and marine clay (F8 = CH; t.t. 3).

*The locality of the third case study* (Fig. 3c) was characteristic of eolian loess loam (soil type F6 = CL; workability class—t.t. 2), fluvial gravel loam (F1 = MG; t.t. 4) and fluvial clay (F6 = CL; t.t. 3), where the last two layers were excavated only partially. Among the non-excavated layers were fluvial gravel-sand (G3 = G-F; t.t. 2), fluvial sand (S3 = S-F; t.t. 2) and fluvial clayey gravel (G5 = GC; t.t. 3).

*In the locality of the fourth case study* (Fig. 3d) the excavated layers were made up by eolian loess loam (soil type F6 = CL; workability class—t.t. 2), proluvial gravel loam (F1 = MG; t.t. 3) and proluvial clay (F6 = CL; t.t. 3). The last layer was excavated only partially. The remaining non-excavated subsoils layers were made up by proluvial sand (S3 = S-F; t.t. 1), proluvial clay (F8 = CH; t.t. 3) and marine sand (R3-5; t.t. 4).

*The locality of the fifth case study* (Fig. 3e) was characteristic of eolian loess loam (soil type F6 = CL; workability class—t.t. 2), anthropogenic backfill (Y with the character of F8 = CH; t.t. 3) and fluvial clay (F8 = CH; t.t. 3), where the last layer was excavated only partially. The non-excavated subsoil layers were fluvial sand (S3 = S-F; t.t. 1), fluvial gravel-sand (G3 = G-F; t.t. 2) and marine clay (F8 = CH; t.t. 3).

*In the locality of the sixth case study* (Fig. 3f) the excavated layers were anthropogenic backfill (soil type Y(G5 = GC); workability class—t.t. 3), eluvium of weathered pelites (R5; t.t. 4), eluvium of slightly weathered pelites (R5; t.t. 5) and marine healthy pelites (R2-4; t.t. 6). The last two layers were both in the excavated and non-excavated part.

## Assessment of soil and rock workability

**Figure 4:**
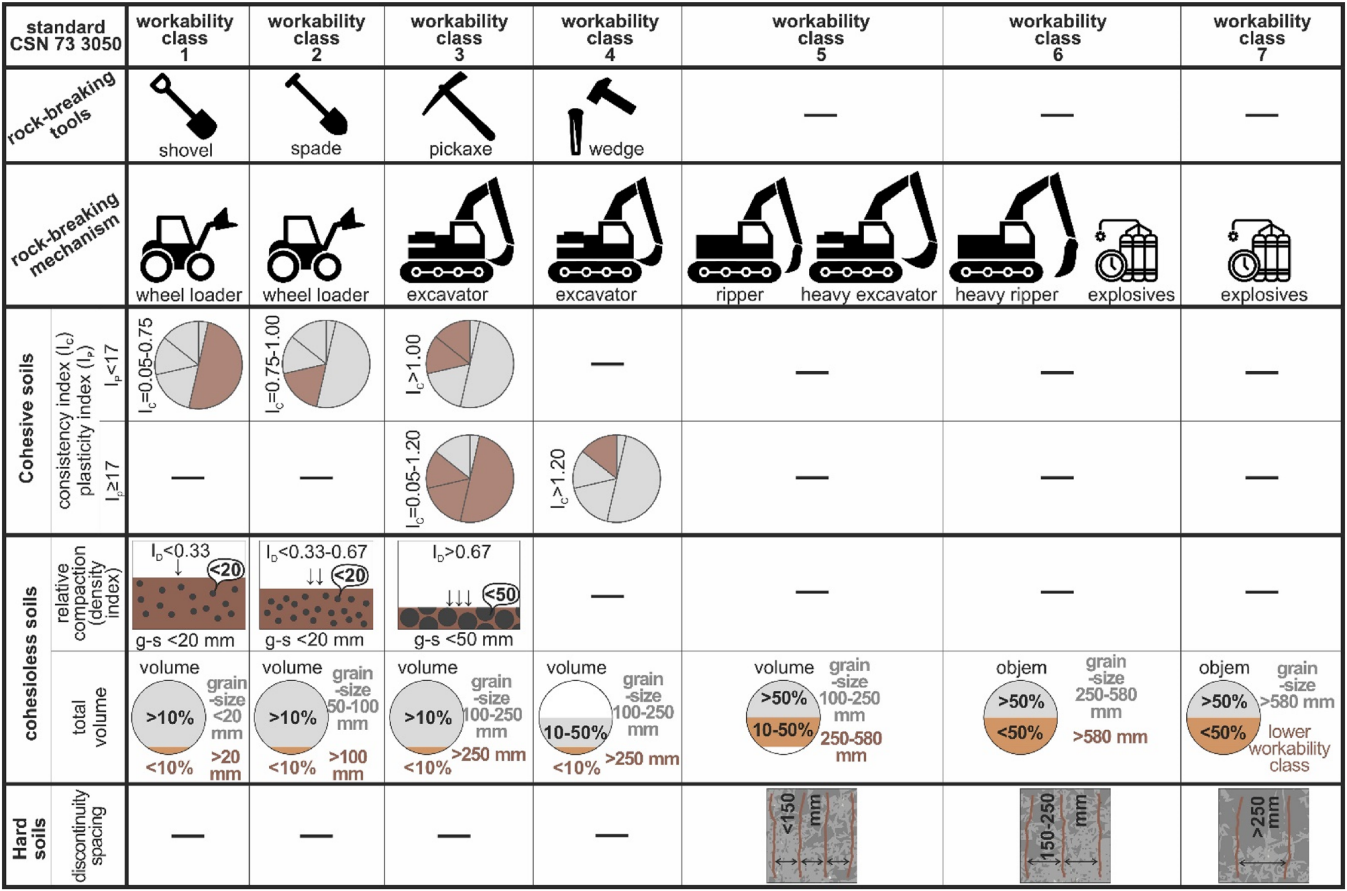
Classification table of soil and rock workability classes.

The first assessed factor was workability classes (Fig. 4) that are in the case studies evaluated using the classification model of according to Standard CSN 73 3050^15,16^. These are rock classification systems used for earthwork during construction work and serve for earthwork pricing. However, apart from the price, they also influence the construction project and earthwork realization.

Soils and rocks are divided into *7 workability classes* (Fig. 4). Various classification criteria are used there, and they are predominantly related to the fact whether it is the case of fine-grained or coarse-grained soils, semi-rock or hard rocks. Workability classes are used to document all engineering-geological boreholes, excavations, earthwork pricing, but also to choose suitable mechanisms for earthwork.

The first group are *cohesive soils* (Fig. 4). Cohesive soils are classified into workability classes based on plasticity index and consistency index. Workability classes 1, 2 and 3 in fine-grained soils mean that plasticity index is below 17, while consistency index is 0.05–0.75 for workability class 1 (soil can be worked by a shovel), 0.75–1.00 for workability class 2 (soils can be worked with a spade) or consistency index over 1.00 for workability class 3 (soil can be loosened by a pickaxe). Or, in case fine-grained soils have a plasticity index equal to or higher than 17 and the consistency index 0.05–1.20, such soils are ranked into workability class 3. In case the consistency index is over 1.20, it is workability class 4.

In *cohesionless soils* we distinguish 2 groups of criteria (Fig. 4). *In the first group* we have the combination of relative compaction (density index) and grain-size distribution. If the density index is below 0.33 and grains are smaller 20 mm, it is workability class 1. If the density index is from 0.33 to 0.67, and grain-size is smaller than 20 mm, it is workability class 2. The third option are cohesionless soils of density index over 0.67, and grain-size distribution below 50 mm, which is workability class 3.

*In the second group of criteria,* it is the case of a combination volume percentage and certain grain-size distribution. Workability class 1 is characterized by grain-size below 20 mm and volume percentage over 10%. The remaining volume percentage below 10% is characterized by grains over 20 mm. The same applies for workability classes 2 and 3—the majority volume percentage over 10% is made up by grains 20–50 mm (workability class 2) and 50–100 mm (workability class 3). The minority volume percentage (below 10%) is constituted by grain-size over 50 mm (workability class 2) and over 100 mm (workability class 3). In workability class 4, the volume percentage below 10% is characterized by grain-size over 250 mm, volume percentage 10–50% for grain-size distribution 100–250 mm, and the remaining volume is characterized by lower workability class 4. The workability class 5 is characterized by gran-size distribution of 250–580 mm (0.1 m^3^) mm and volume percentage 10–50%, and grain-size 100–250 mm in the volume percentage over 50%. A similar principle of volume percentages applies in workability classes 6 and 7, where the workability class 6 has grain-size distribution of 250–580 mm (0.1 m^3^) for a larger percentage volume (over 50%), while workability class 7 is characterized by grain-size distribution below 580 mm (0.1 m^3^). Smaller volume percentage (below 50%) is characteristic of grain-size below 580 mm (0.1 m^3^) in workability class 6, and workability class 7 it is expressed by the remaining occurrence of hard rocks of lower workability class than 7. The grain-size mean of 580 mm has the value 0.1 m^3^ in the standard.

The occurrence of *hard rocks* (Fig. 4) is typical for higher workability classes as the higher strength of rocks mean they are not easily broken or loaded. The assessment criterion is discontinuity spacing. The workability class 5 is characterized by discontinuity spacing below 150 mm. In the workability class 6 the discontinuity spacing is 150–250 mm, and in workability class 7 it is over 250 mm.

The least important criterion for workability class assessment is the use of manual tools or machinery. *Manual tools* (Fig. 4) are used only in the first four workability classes, where workability class 1 is workable by a shovel. In workability class 2 we need to use a spade, and in class 3, we need to use a pickaxe. Ruling out shovels, spades or pickaxes, in workability class 4 we need to employ a wedge and sledgehammer. For the remaining workability classes, we need to use machinery.

As for *machinery* (Fig. 4) in connection with workability classes, there are the following rules. For workability classes 1 and 2 we can use a wheel loader, for workability class 3 and 4 we use an excavator, and for workability class 5 we use a ripper or a heavy excavator, or explosives. For workability class 6 we use a heavy ripper or explosives. For workability class 7 only explosives are used. In line with technological progress, new mechanisms may be applied for the different workability classes. At the same time, for some workability classes we use identical mechanisms, but the difference is in the spent energy on loosening and loading. Also, there will be different extent of wear. All this must be reflected in the selected workability class and corresponding price of earthwork.

### Assessment of soil and rock workability based on the price of 1 m^3^ of excavated soil

Out of all the properties we assess within engineering-geological investigations, the most economically significant^24,25^ for construction works is soil and rock workability as it is used for earthwork pricing. This mainly applies in construction projects with dominant volume of earthwork. In sewer system construction projects, earthwork plays the decisive role.

**Figure 5:**
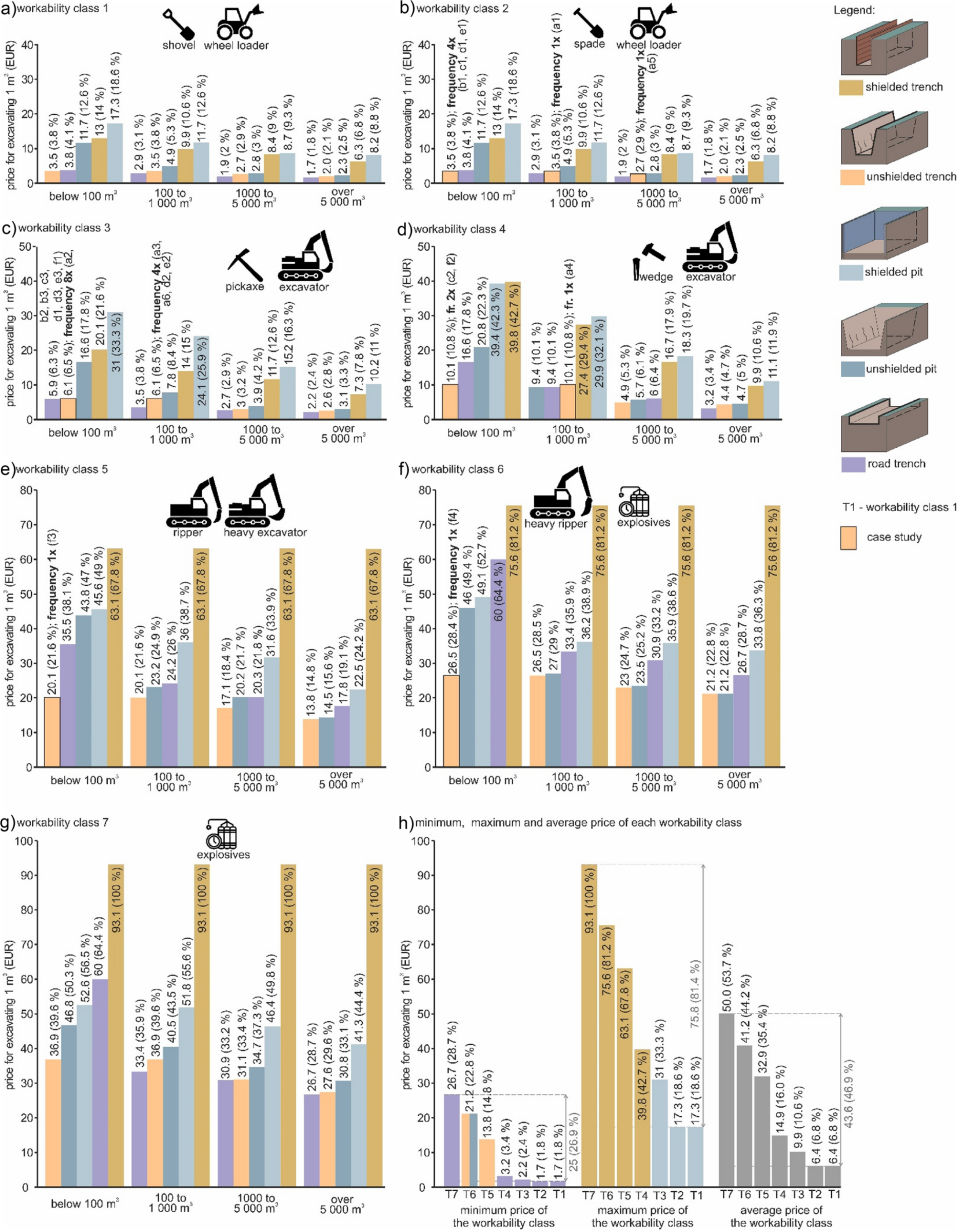
Graphs of the workability class prices (1–7) and a summarized graph; (**a**) workability class 1; (**b**) workability class 2; (**c**) workability class 3; (**d**) workability class 4; (**e**) workability class 5; (**f**) workability class 6; (**g**) workability class 7; (**h**) minimum, maximum and average price of each workability class.

The first assessment criterion is the *influence of a particular workability class on the price of 1 m*^*3*^ of earthwork. We produced graphs for each workability class (Fig. 5a–h), where the last Fig. 5h shows the minimum, maximum and average values of all graphs describing the workability class prices.

The first and second graphs of workability classes 1 and 2 (Fig. 5a,b) show the lowest prices when compared to the remaining ones. This is logical because workability classes 1 and 2 have the easiest breakability. The prices in the two workability classes are identical due to a somewhat conventional averaging of prices in the two classes.

As for *workability class 1 and 2 costs* (Fig. 5a,b), it showed that the minimum cost of earthwork is Eur 1.7 per 1 m^3^, which corresponds to 1.8% of the maximum price of all workability classes. This is because the simplest excavation technologies are used (road trench). Another reason is the highest volumes (over 5000 m^3^), which are always cheaper (in higher volumes, machinery and staff are already on site, which makes the price of 1 m^3^ cheaper). The calculation of percentages is made up by comparing the maximum price out of all the workability classes. On the contrary, the maximum price of earthwork in workability classes 1 and 2 is Eur 17.3 (18.6%), which is related to the most demanding excavation technology (shielded pit) and the lowest cubic volume (below 100 m^3^). The average price is Eur 6.4 (6.8%).

*Workability class 3* (Fig. 5c) is priced at Eur 2.2 (2.4%) per 1 m^3^, which means a rise of 0.6% in comparison with the two previous workability class minimum prices. Similarly to the previous case it was a road trench of high cubic capacity (over 5000 m^3^). On the contrary, the maximum price in this workability class is Eur 31.0 (33.3%) with an increase of 14.7%. Also, in this case a similar technology (shielded pit) was used, and the cubic capacity was below 100 m^3^. The average price was Eur 9.9 (10.6%).

The minimum price of *workability class 4* (Fig. 5d) rose by 1.0%, when compared to the previous workability class, to Eur 3.2 (3.4%) per 1 m^3^. The maximum value rose by 9.4–42.7% (Eur 39.8). As for the correlation of price minimum and maximum related to excavation technology and cubic volumes, the relationship is compatible with the previous cases; only in the maximum price the excavation technology of shielded pit changed to shielded trench. This category has an average cost of Eur 14.9 (16.0%) within whole workability class 4.

In connection with the minimum price of *workability class 5* (Fig. 5e) there was the highest increase (11.4%), when compared to the previous class, to 14.8% (Eur 13.8 per 1 m^3^). As for the maximum price, there was also an increase in comparison with the previous workability class 4 (of 25.1%) to 67.8% (Eur 63.1). As for the correlation with the previous cases, the technology changed for the minimum price. Instead of road trench, the technology was unshielded trench. Talking of the maximum price, it is similar to the previous case—instead of the shielded pit, shielded trench was used. As for the cubic volume, the minimum price is related to volumes over 5000 m^3^ and maximum price differs to previous cases (all volumes—below 100 m^3^, 100–1000 m^3^, 1000–5000 m^3^, over 5000 m^3^), while in workability classes 1–4 the cubic volume was always below 100 m^3^. The average price in this class is Eur 32.9, and thus represents 35.4% of the maximum price in all workability classes.

In *workability class 6* (Fig. 5f) the minimum price is Eur 21.2 (22.8%) per 1 m^3^, which corresponds to an increase of 8.0% when compared with workability class 5. The minimum price is this workability class is identical to the previous one (unshielded trench), but the minimum price also concerns unshielded pit. The maximum price is Eur 75.6 (81.2%) and corresponds to a rise of 13.4%. The same technology is used, i.e., shielded trench. As for the volume, the situation is identical to the previous case, both as for the minimum and maximum price. The average value is Eur 41.2, which is 44.2%.

*The workability class 7* (Fig. 5g) is priced at Eur 26.7 (28.7% for road trench) per 1 m^3^, which constitutes a rise of 5.9% when compared with the minimum price of the previous workability class. As for technology, road trench was used. The maximum price in this workability class is Eur 93.1 for shielded trench, which is 100% of the maximum price for all the workability classes (an increase of 18.8% when compared with workability class 6). As for the excavation technology, the maximum price did not change. As for the cubic volume, the correlations of the minimum and maximum price remained unchanged. In the minimum values, the earthwork cubic volume is below 5000 m^3^ and in the maximum value, the cubic volume is below 100 m^3^, 100–1000 m^3^, 1000–5000 m^3^, or over 5000 m^3^. The average price is Eur 50.0 (53.7%).

Figure 5 gives the frequencies of the 6 case studies, in which the column price is complemented with frequencies and order number of assessed layers. This means that the column with a price is visually highlighted in unshielded trench, which was implemented in the case studies. The case studies will be described in detail below.

**Figure 6:**
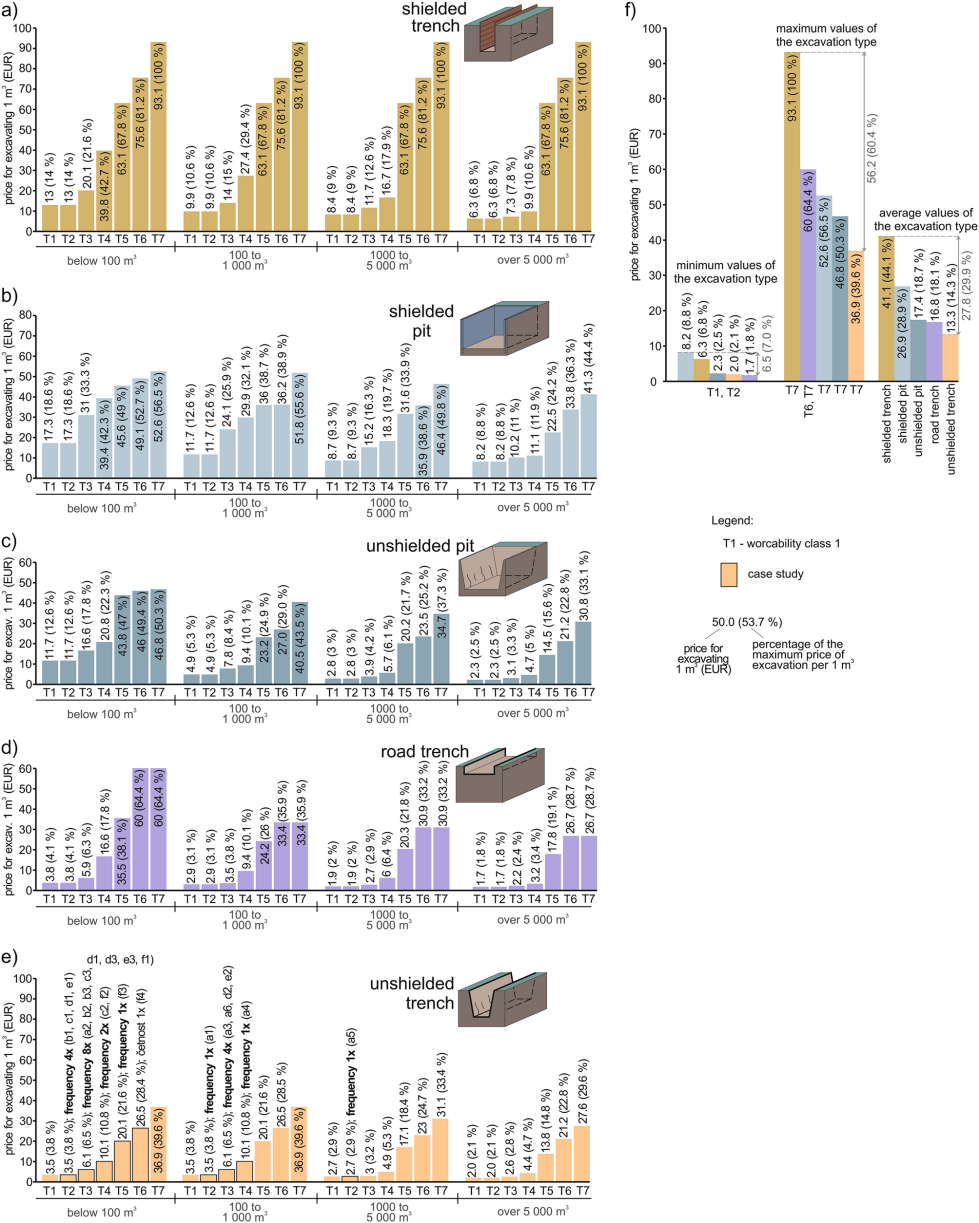
Graphs of the different types of excavations and technologies used; (**a**) shielded trench; (**b**) shielded pit; (**c**) unshielded pit; (**d**) road trench; (**e**) unshielded trench; (**f**) minimum, maximum and average price of each excavation type and technology.

The second assessed criterion (factor) will be the *influence of the excavation technology* (Fig. 6a–f). To produce Fig. 6, data from Fig. 5 were used, but Fig. 6 is presented separately to independently assess the factor mentioned above.

Comparing all the five technologies (shielded trench, unshielded trench, shielded pit, unshielded pit and road trench) it shows that the *most costly excavation type is *shielded trench (Fig. 6a). It is important to take into account that the price reflects the costs, and the costs reflect the factors at play. Shielded trench has a linear character, and when excavating it, the performance of the machinery cannot be optimised as in the case of other types of excavations with a more spatial character. It is logical that in this most expensive technology, the workability class 7 is the most costly (at cubic volume below 100 m^3^) as this concerns the geological structure that is most difficult to loosen and load. On the contrary, the cheapest is the workability class 1 and 2, which are the easiest to loosen and load (at cubic volumes over 5000 m^3^). The ratio between the cheapest and most expensive workability class is 15-fold (93.1/6.3 = 14.8).

The second most costly excavation type is *shielded pit* (Fig. 6b). Using shielded pit, it is more likely to optimise the gradual excavation spatially. The most costly is workability class 7 at cubic volumes below 100 m^3^, and the cheapest workability classes 1 and 2 at cubic volumes over 5000 m^3^. The ratio of the cheapest and most expensive option is sixfold (52.6/8.2 = 6.4).

The third most costly excavation type is *unshielded pit* (Fig. 6c). Supports need not be erected when compared with the previous technology. Aa for the most expensive and cheapest value, the situation is identical to shielded pit, but the ratio is 20-fold (46.8/2.3 = 20.4).

*Road trench* is the fourth most expensive type of excavation (Fig. 6d). It is relatively low demanding and easy to optimise when compared with the previous excavation types. The ratio of the cheapest and most expensive option is the highest (35-fold; 60.0/1.7 = 35.3). This means that the engineering-geological structure is the most important in this type of excavation (apart from cubic volume). The difference is caused by the differences between the most expensive workability classes 6 and 7 (at cubic volume below 100 m^3^) and the cheapest workability classes 1 and 2 (at the cubic volume over 5000 m^3^).

The least expensive type of excavation is *unshielded trench* (Fig. 6e), in which the costs are reduced by the absence of trench supports. The ratio between the minimum price (workability class 7 and cubic volume below 100 m^3^ and 100–1000 m^3^) and the maximum price (workability class 1 and 2 at the cubic volume over 5000 m^3^) is 19-fold (36.9/2.0 = 18.5), which points at the importance of the geological structure.

**Figure 7:**
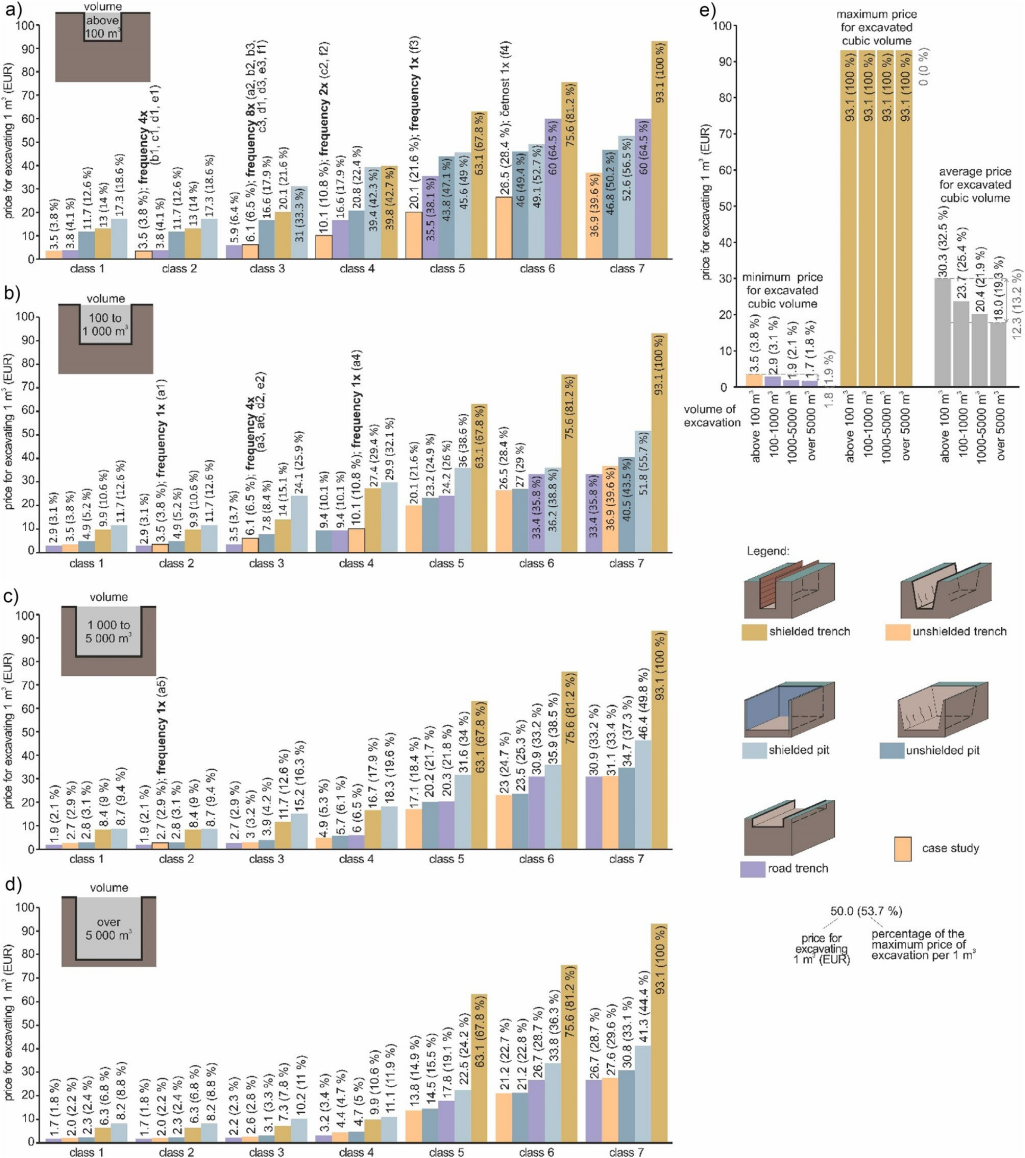
Graphs of assessed excavated cubic volumes; (**a**) excavated cubic volume below 100 m^3^; (**b**) excavated cubic volume 100–1000 m^3^; (**c**) excavated cubic volume 1000–5000 m^3^; (**d**) excavated cubic volume over 5000 m^3^; (**e**) maximum, minimum and average price of excavated cubic volumes.

The third assessed criterion (factor) is the *influence of excavated cubic volume per price of 1 m*^*3*^ within earthwork. We produced four graphs with four excavated cubic volumes, namely below 100 m^3^ (Fig. 7a), 100–1000 m^3^ (Fig. 7b), 1000–5000 m^3^ (Fig. 7c) and over 5000 m^3^ (Fig. 7d). At the same time, Fig. 7e shows the minimum, maximum and average prices with respect to the four excavated cubic volumes.

As for the assessment of *cubic volume below 100 m*^*3*^ (Fig. 7a) there is a clear dependency in the sense that the prices rise from the cheapest workability classes 1 and 2 to the most expensive workability class 7 in each type of excavation and technology. It is also clear that the lowest price at such cubic volume is in workability classes 1 and 2 using unshielded trench. On the other hand, the most expensive is the complicated technology of shielded trench. In all workability classes, the prices rise in the order: unshielded trench, road trench, unshielded pit, shielded trench and shielded pit). There are only three exceptions. In workability class 3 the most costly technology is shielded pit and the next-to-last is shielded trench. The order of these two types changes in workability classes 4 and 5. In workability classes 6 and 7, the second most expensive technology is road trench because of reduced capacity to optimise earthwork (linear excavations are more difficult to optimise in hard rocks).

When comparing the cubic volume below 100 m^3^ (Fig. 7a) with other cubic volumes (100–1000 m^3^; Fig. 7b, 1000–5000 m^3^; Fig. 7c and over 5000 m^3^; Fig. 7d), the order is the almost identical. The rule is that the prices rise along with an increase in the workability class and more demanding technology of excavation.

When comparing the *ratio between the price minimum and maximum* (Fig. 7e) in connection with the four assessed cubic volumes, it shows, for example, the ratio between the minimum and maximum at the smallest cubic volume below 100 m^3^ (Eur 89.6), which constitutes a 27-fold ratio (93.1/3.5 = 26.6). In other volumes, the ratio is even up to 55-fold (93.1/1.7 = 54.8) at cubic volume over 5000 m^3^. This factor is clearly important. If we assess the average prices for the excavated cubic volumes, the ratio between the most expensive price for cubic volume below 100 m^3^ and the minimum average price for excavated cubic volume over 5000 m^3^ is Eur 12.3 (13.2%).

The following text will describe the results of the influences of all important parameters that participated in the implementation of earthworks and are therefore also part of their pricing. Therefore, in the final result, this influence is reflected in the cost of earthworks. For many buildings, earthworks are one of the most important items in the total construction costs. Especially it concerns those structures that work with large volumetric changes, such as the displacement of rock masses (soil or rock). The *influence of the workability class* will be evaluated first because this parameter reflects the amount of work needed to break and load rock masses. This means that more easily breakable rocks (such as soil) will have a smaller share of the total price than harder-to-breakable rocks such as rock. The second evaluated influence is the *influence of excavated cubic volume*. Here, more volume cubic meters will have a greater impact on the overall price than smaller cubic meters. However, this amount is also considered in the fact that one cubic meter will be cheaper in the total amount for more volume than for fewer volume earthworks. The third evaluation will be the *influence of the type of excavation and its technology*. Such as unshielded trench, road trench, shielded pit, unshielded pit, and shielded trench. In this case, simpler and less demanding types of excavation and their technology are cheaper than more complex and demanding types (in the previous sentence they are sorted ascending according to this statement).

**Figure 8:**
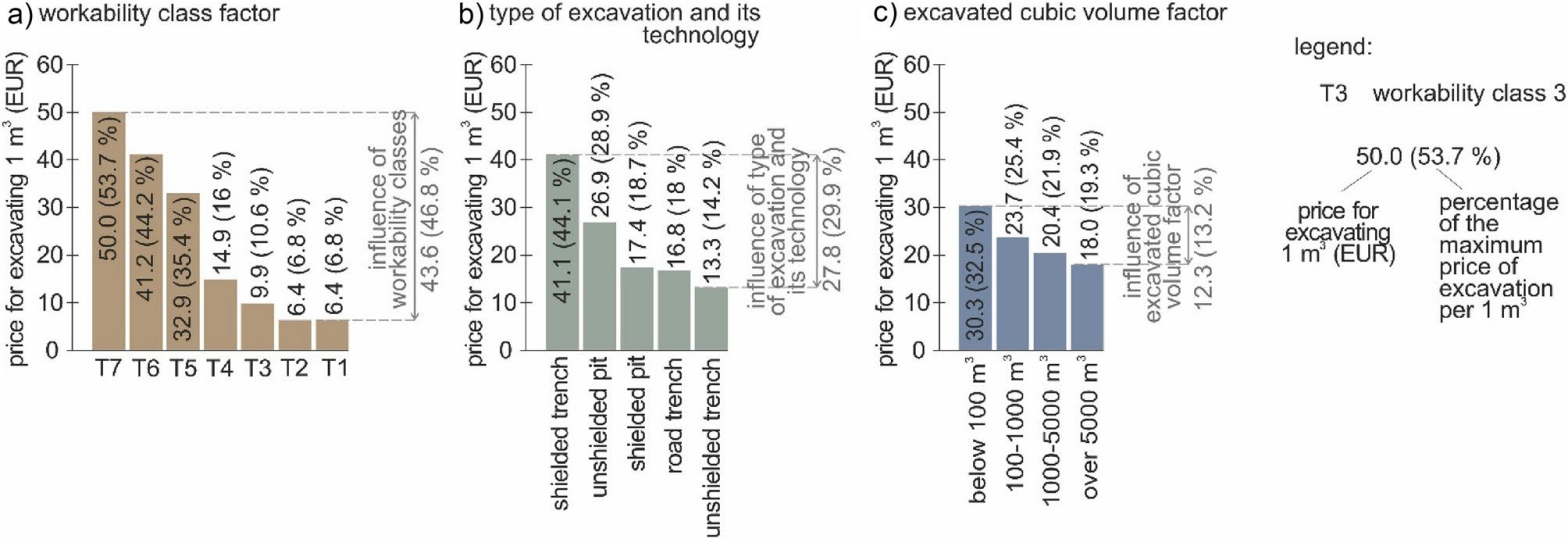
Graphs of the influence of the different earthwork factors on the price of 1 m^3^ earthwork (evaluation approach—study 1); (**a**) workability class factor; (**b**) type of excavation and its technology; (**c**) excavated cubic volume factor.

To compare the different factors (workability classes, excavated cubic volume and type of excavation and its technology) affecting the price of 1 m^3^ of earthwork, we used the comparison of average prices and the factors (Fig. 8). The most influence on the pricing was observed with the engineering-geological structure represented by workability class, i.e., 46.8% (Eur 43.6; Fig. 8a). The influence was calculated as a percentage ratio between the minimum and maximum average price of the lowest and highest workability classes. The second most prominent influence was observed with the type of excavation and its technology, i.e., 29.9% (Eur 27.8; Fig. 8b). This influence was obtained as a percentage ratio between the minimum and maximum price of the cheapest and most expensive technologies. The third was the excavated cubic volume, i.e., 13.2% (Eur 12.3; Fig. 8c). This was calculated as a percentage ratio between the minimum and maximum average price of the cheapest cubic volume category over 5000 m^3^ and the most costly cubic volume category below 100 m^3^.

When we compare all these influences, it shows that the influence of 46.8% in engineering-geological structures represented by workability classes has almost double (1.6) influence than the type of excavation (29.9%). Therefore, when planning earthwork, it is most important to pay attention to engineering-geological investigations to determine the geological structure precisely as for workability classes. This has a fundamental influence on the determination of earthwork prices (46.8%). The remaining part of the price is determined by the type of excavation and its technology (29.9%) and excavated cubic volumes (13.2%).

### Assessment of workability classes based on overall excavated cubic volume price in the 6 case studies

**Figure 9:**
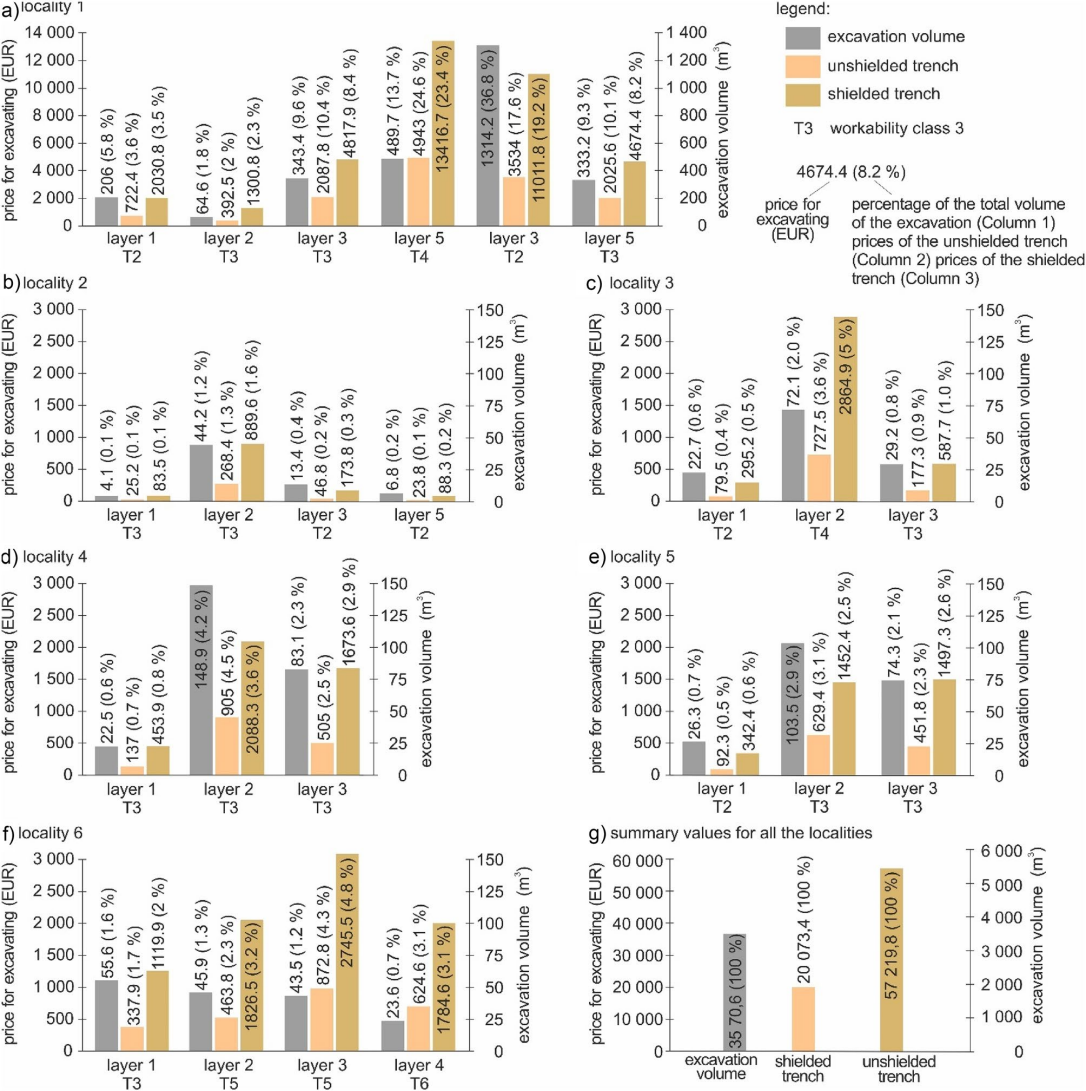
Graph comparing the excavated cubic volumes (m^3^) in the different case studies and the price (EUR) using the technology of unshielded trench (evaluation approach—study 2a) and shielded trench (evaluation approach—study 2b); (**a**) locality 1; (**b**) locality 2; (**c**) locality 3; (**d**) locality 4; (**e**) locality 5; (**f**) locality 6; (**g**) summary values for all the localities.

**Figure 10:**
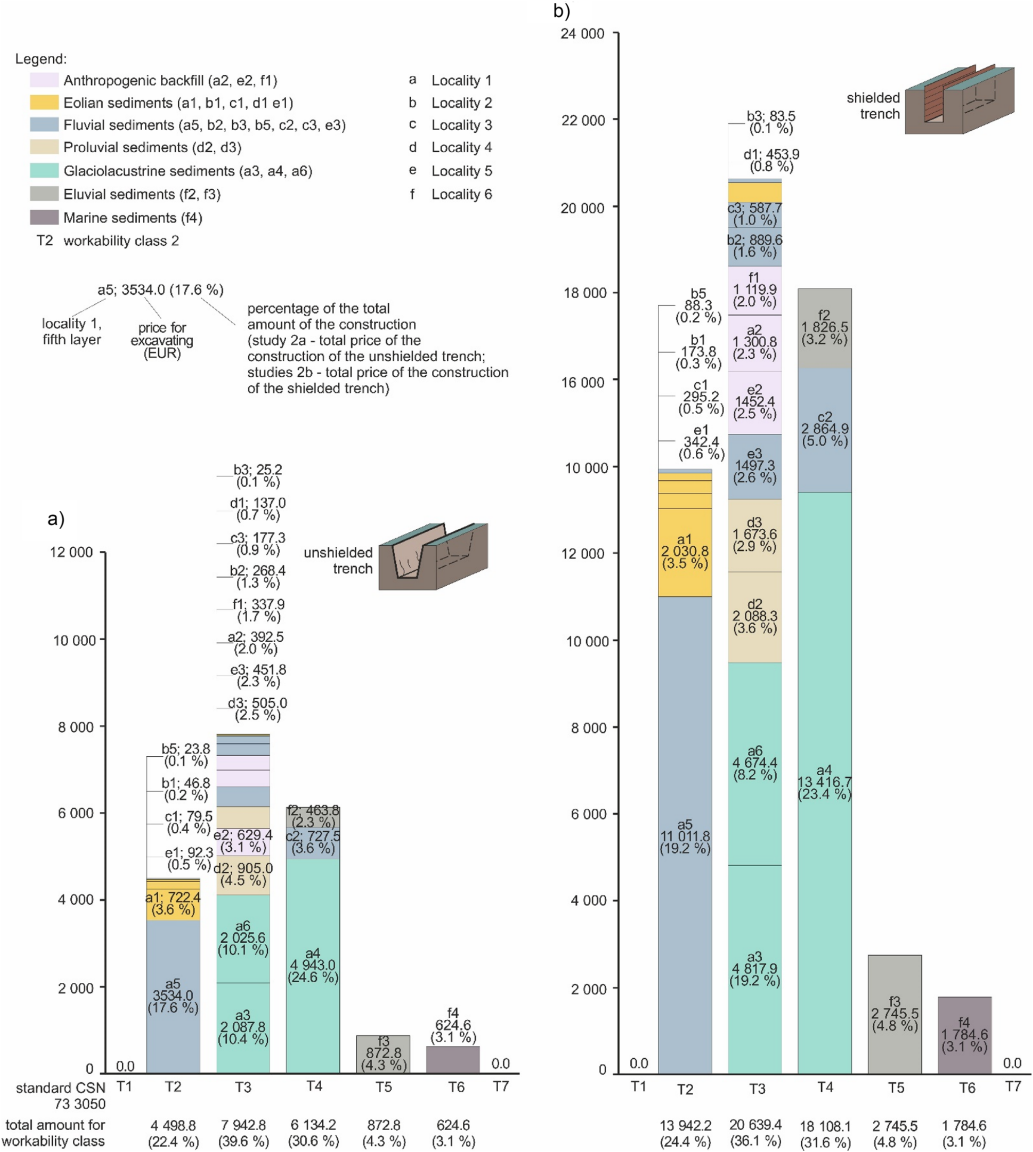
Graph of total prices for all case studies in dependence on workability classes and different layers, including their genesis; (**a**) the implemented option 2a—unshielded trench; (**b**) hypothetical option 2b—shielded trench.

The six sewer system case studies assessed in engineering-geological sections and described in this subsection (Fig. 3) are localized on the geological map Fig. 2. The different sewer systems were technologically implemented as unshielded trench (Study 2a), while their pricing is given per each locality in Fig. 9a–f. The pricing is summarized in Figs. 9g and 10. Each sewer system 1–6 (case study) was implemented using the technology of unshielded trench but there is also a calculation for the technology of shielded trench (Study 2b), which was not implemented.

When we compare the case studies (Fig. 9a–f), the highest excavated cubic volume was in the first locality, i.e., 77.0% (2751.1 m^3^) out of all the localities. Although *the first case study* (Fig. 9a) was implemented in 6 layers, there were only three workability classes (2, 3 and 4). The most dominant was the fourth layer (489.7 m^3^), which represented Eur 4943.0 (24.6%) in unshielded trench. The second option of shielded trench was 2.7 times more expensive (Eur 13,416.7).

*The second case study* (Fig. 9b) represented 1.9% (68.5 m^3^) of all excavated cubic volume in all case studies. It was implemented in four layers and two workability classes (2 and 3). The most voluminous was the second layer from the ground surface (44.2 m^3^), which amounted at Eur 268.4 (1.3%) using the technology of unshielded trench. The non-implemented technology of shielded trench was 3.3 times more expensive (Eur 889.6).

When compared with all the assessed localities, the* third case study* (Fig. 9c) represented 3.5% (124.0 m^3^) of the excavated cubic volume. In this case study, only three layers were assessed (workability classes 2, 3 and 4). The most voluminous (72.1 m^3^) was the second layer with the price for unshielded trench of Eur 727.5 (3.6%) and for shielded trench of Eur 2864.9 (5.0%). This means that shielded trench was 3.9 times more expensive than unshielded trench.

The *fourth case study* (Fig. 9d) represented 7.1% (254.5 m^3^) of the excavated cubic volume out of all the localities. The fourth case study was implemented in 3 layers as above, but only under one workability class (3). The most voluminous was the second layer from the ground surface (148.9 m^3^). This volume costs Eur 905.0 (4.5%) to be excavated using the technology of unshielded trench, and Eur 2088.3 if the technology of shielded trench was used (2.3 times more expensive).

*The fifth case study* (Fig. 9e) represents 5.7% (204.1 m^3^) of all the excavated cubic volume. Three layers of two workability classes (2 and 3) were excavated. The most voluminous was the second layer (103.5 m^3^), which cost Eur 629.4 (3.1%) in unshielded trench and Eur 1452.4 in shielded trench (2.3 times more expensive).

As for the total excavated cubic volume, the *sixth case study* (Fig. 9f) represents 4.7% (168.6 m^3^). Four layers were assessed there of workability classes 3, 4, 5 and 6. The most voluminous was the first layer (55.6 m^3^), which cost Eur 337.9 (1.7%) in unshielded trench, and Eur 1119.9 (2.0%) in shielded trench. It is interesting that in this case study, the first layer (workability class 3) had the highest volume but cost the least to excavate when compared with higher workability classes. This did not occur in other case studies.

The overall excavated cubic volume in all the six localities was 3570.6 m^3^ (Fig. 9g). The total price was Eur 20,073.4 using the technology of unshielded trench, while the hypothetical option would cost Eur 57,219.8 (2.9 time more).

If we assess all the case studies in one graph (Fig. 10), it is possible to observe the following. Out of the seven workability classes, we managed to identify only five in the six studied localities (workability classes 2, 3, 4, 5 and 6), leaving thus classes 1 and 7 out. The most abundant was workability class 3 with 39.6% (Eur 7 942.8 for unshielded trench; Fig. 10a). Interestingly, this class is made up by the highest number of genetic types of soil (glaciolacustrine, proluvial, anthropogenic, fluvial and eolian sediments), while glaciolacustrine sediments dominated (20.5%). The second most abundant class was workability class 4 with 30.6% (Eur 6134.2) constituted by 3 different genetic types (glaciolacustrine, fluvial and eluvial sediments). The third came workability class 2 with 22.4% (Eur 4498.8) with 2 genetic types (glaciolacustrine and eolian sediments). All the three most dominant classes were most abundant for the genetic type of glaciolacustrine sediments. The last two workability classes 5 (4.3%) and 6 (3.1%) only had a small share, and were represented by eluvial and marine sediments.

For better visibility and comparison, we made a graph for the pricing of shielded trench (Fig. 10b) too. We may observe changes in the total prices in relation to the workability classes in all case studies, and at the same time, there are also the different layers and their genesis. The ratio of the total price of study 2a using the technology of unshielded trench is 2.9 times cheaper than in study 2b using the technology of shielded trench.

**Figure 11:**
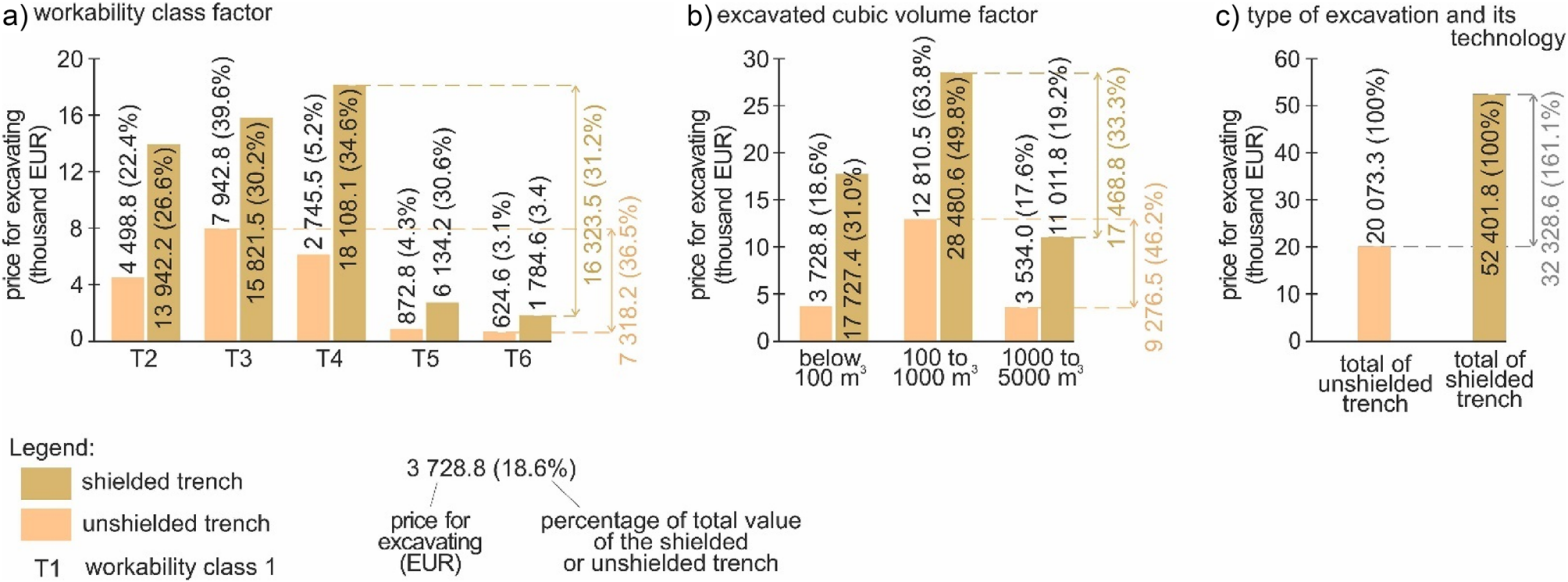
Graphs of different earthwork factor influence on the price of excavations (study 2a—unshielded trench, the first column; study 2b—shielded trench, the second column), (**a**) workability class factor; (**b**) excavated cubic volume factor; (**c**) type of excavation and its technology.

In conclusion of this Section (Studies 2a and b), we identified the influence of the three examined factors on the price of the sewer system earthwork implementation. If we assess the influence of workability classes, average prices were used for the assessment. Figure 11 gives the graphs comparing the excavated cubic volumes and their prices in the 6 case studies as a sum.

The first assessed factor was the workability classes (Fig. 11a). We found that the most dominant classes (for unshielded trench—study 2a) were workability classes 2 (22.4%), 3 (39.6%) and 4 (13.7%). On the contrary, the least dominant were workability classes 5 (4.3%) and 6 (3.1%). The ratio between the minimum and maximum average price was 36.5% considering the technology of unshielded trench. There is an analogy with the second option of shielded trench (study 2b) but the ratio between the minimum and maximum average price amounted to 31.2%. Clearly, the extreme workability classes (1 and 7) were not excavated within the 6 case studies and thus were not included in the price.

The second assessed factor was excavated cubic volume (Fig. 11b). The most dominant group (in unshielded trench—study 2a) was cubic volume from 100 to 1000 m^3^ (63.8%) and, the least dominant group are excavated cubic volume from 1000 to 5000 m^3^ (17.6%). The difference in the average price between the cheapest and most expensive cubic volume was 46.2%. The order of the assessed cubic volumes for shielded trench (study 2b) was identical to the second assessed group of unshielded trench (study 2a), while the difference between the cheapest and the most expensive cubic volume was 33.3% considering the average prices. The cubic volume over 5000 m^3^ is missing as it made part of the study 1 only.

The third assessed factor was to compare the implemented technology of unshielded trench with the cost of shielded trench (Fig. 11c). The difference in the average prices of the two technologies was 161.1%.

**Figure 12:**
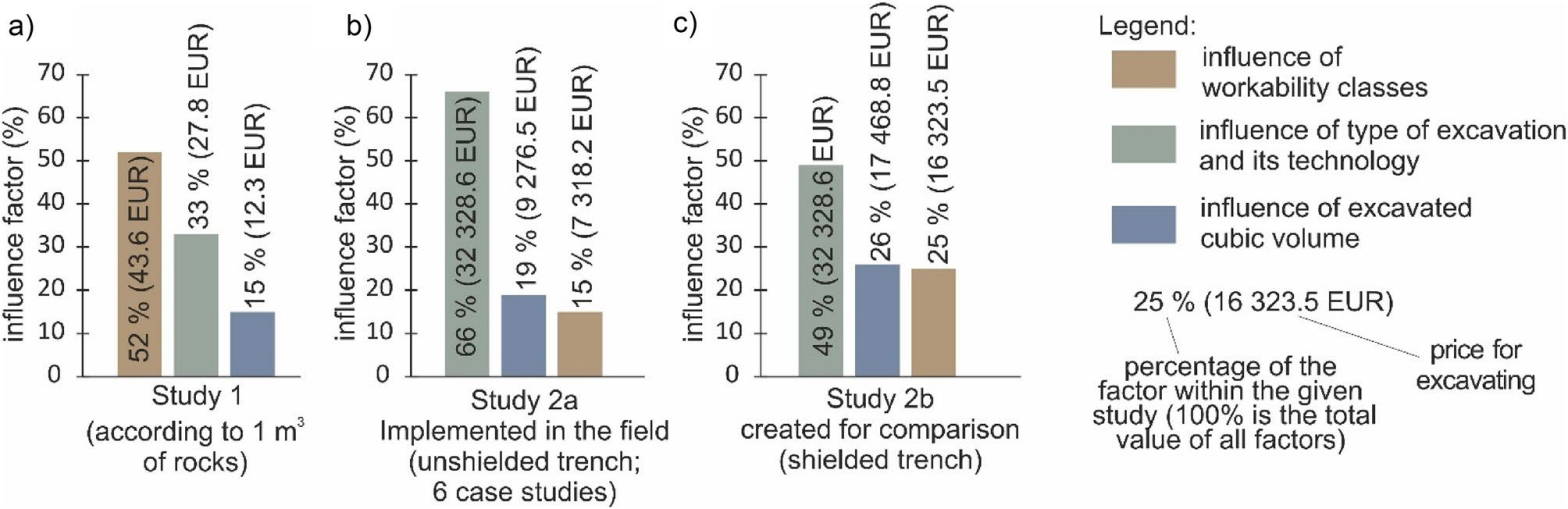
Graph of study results with quantified levels of influence of the different factors on the price of earthwork (workability class, type of excavation and its technology, excavated cubic volume), (**a**) study 1; (**b**) study 2a; (**c**) study 2b.

In conclusion of studies 2a and 2b, we can state we identified a structured influence of factors (Fig. 12). To compare the results of the second study, we also state the results of study 1 (Fig. 12a). As for the second study, in the technology of unshielded trench (study 2a) the most decisive factor was the type of excavation and its technology with 66% (Fig. 12b); the second factor was the excavated cubic volume with 19%, and the third was the influence of workability class with 15%. In the technology of shielded trench (Fig. 12c; study 2b) the influence was analogous: 49% (the type of excavation and its technology), 26% (excavated cubic volume) and 25% (workability class).

These results are affected by the different number of types of excavations. In study 2, we compared only two technologies (shielded and unshielded trench), while in the study 1, we compared five types of excavations. Next, the 6 localities (case studies) did not include the extreme workability classes 1 (loose, unconsolidated soils workable by a shovel) and 7 (healthy hard rocks). The last reason is that the cubic volume over 5000 m^3^ was not assessed in the case studies. Having combined these boundary conditions, the factors were influenced significantly.

## Conclusion

We carried out two studies (evaluation approaches) assessing the influence of different factors (workability class, type of excavation and its technology, and excavated cubic volume) on the price of earthwork. For study 1, we used the assessment unit of 1 m^3^ of excavated soil. Study 2 was assessed on the basis of 6 case studies, where different factors were assessed based on the real volumes of earthwork, real volumes of excavated and loaded layers and their workability classes. All these factors were taken as the representatives of engineering-geological influence on the price of earthwork. At the same time, we assessed the influence of the implemented type of excavation and its technology (unshielded trench—study 2a), which was compared with a more complicated technology (shielded trench—study 2b).

In *study 1,* we identified the following order of influence on the price of earthwork. The first dominant factor was the workability class with 52%. The second most important factor was the type of excavation and its technology with 33%. The least influence on the price of earthwork was established in the excavated cubic volume (15%), which is somewhat surprising, considering a common concept that the volume belongs among the decisive price factors.

*Study 2* was implemented in two variants (2a and 2b), where the first variant concerned the real excavated sewer system using the technology of unshielded trench in 6 case studies. The second variant was calculated hypothetically for the sake of comparison with study 2a.

The results in *Study 2a* differed to study 1 and had the following order of factors: the highest influence was observed in the type of excavation and its technology (66%), the second came the workability classes (19%) and third was excavated cubic volume (15%). In *Study 2b* we found that the most significant influence was in the type of excavation (49%), second came the excavated cubic volume (26%) and third was workability classes (25%).

The combination of cubic volume and dominance or lack of certain workability classes caused ratio in the factor quantifications. The order of factor influence on the price of earthwork clearly changed when compared with study 1, which allows us to better assess the validity of this research.

Study 1 appears to have a higher validity, where we assessed all the three factors in the full range of all categories. Study 2 had some categories missing, e.g., as for workability classes, there were no workability classes 1 and 7. Also, the category of excavated cubic volume over 5000 m^3^ could not have been included in the quantification. As for the third factor (type of excavation and its technology), we compared only two categories (unshielded trench and shielded trench). Based on the above mentioned, the validity of study 2 is lower. However, it is very rare that a locality has all categories. Study 2 is valuable because these are real-life localities (case studies), which is important for such assessment. Despite the results, it is the methodology that is important. Such methodology may be applied in other localities.

Considering the fact that earthwork represents one of the most dominant groups of work in the construction industry, this study is important for engineering geology, geotechnics and whole construction industry because of the *structured attention to each assessed factors of earthwork*.

The established influences and quantifications may be used for research and teaching purposes to inform all groups of experts what to place impact on when documenting and mapping building localities in order to determine workability classes correctly. The different factors determine the total costs of earthwork, while the costs of earthwork influence the price of a construction project. An important finding is that the correct identification of workability classes has the dominant influence on the price.

## Data Availability

All data analysed during this study are included in this published article.

## References

[1] Griffiths JS, Radford T (2012). An introduction to earthworks in Europe. Geol. Soc. Lond. Eng. Geol. Spec. Publ..

[2] Gunn, D. A., Reeves, H. J., Chambers, J. E., Ghataora, G. S., Burrow, M. P. N., Weston, P., Lovell J.M., Nelder L., Ward D., Smith, R. T. (2008). New geophysical and geotechnical approaches to characterise under utilised earthworks. *Adv. Transp. Geotech*., 313–320. CRC Press.

[3] Herle V (2012). Czech standards and specification for earthworks. Geol. Soc. Lond. Eng. Geol. Spec. Publ..

[4] Meurant G (2013). Soil Mechanics of Earthworks, Foundations and Highway Engineering.

[5] Azahar, M. A., Mahadi, N. F. Z., Rusli, Q. N., Narendranathan, N., Lee, E. C. (2019, April). Use of geophysics for site investigations and earthworks assessments. in *IOP Conference Series: Materials Science and Engineering* (Vol. 512, No. 1, p. 012007). IOP Publishing.

[6] Crapper M, Fell M, Gammoh I (2014). Earthworks risk assessment on a heritage railway. Proc. Inst. Civ. Eng.-Geotech. Eng..

[7] Tsegaselassie A, Tadesse S (2015). Supplementing conventional site investigation techniques of earthworks and sub-grade soils with geophysical investigation in road design. Zede J..

[8] Xu YS, Shen SL, Du YJ (2009). Geological and hydrogeological environment in Shanghai with geohazards to construction and maintenance of infrastructures. Eng. Geol..

[9] Kang GC, Tobita T, Iai S (2013). Damage to sewerage systems during the 2004 Earthquake in Niigata-ken Chuetsu, Japan. Eng. Geol..

[10] Du YJ, Fan RD, Liu SY, Reddy KR, Jin F (2015). Workability, compressibility and hydraulic conductivity of zeolite-amended clayey soil/calcium-bentonite backfills for slurry-trench cutoff walls. Eng. Geol..

[11] Marschalko M, Lahuta H, Juris P (2008). Analysis of workability of rocks and type of prequarternary bedrock in the selected part of the Ostrava conurbation by means of geographic information systems. Acta Montanistica Slovaca.

[12] Marschalko M, Bednárik M, Yilmaz I (2012). Evaluation of engineering-geological conditions for conurbation of Ostrava (Czech Republic) within GIS environment. Environ. Earth Sci..

[13] Marschalko M, Vicherek P, Vicherková M, Yilmaz I, Kubáč J, Popielarczyk D, Kempa T, Yang S (2020). Soil contamination by tar in the alluvial sediments: Case study of the brownfield remediation project in the Czech Republic. Environ. Earth Sci..

[14] Zichella L, Bellopede R, Marini P, Tori A, Stocco A (2017). Diamond wire cutting: A methodology to evaluate stone workability. Mater. Manuf. Process..

[15] Standard CSN 73 3050 (1987) Earthwork. General requirements. Prague: Publishing House of the Office for Standardization and Measurement, Validity: 09/1987–02/2010.

[16] Standard STN 73 3050 Earthwork. General requirements. Slovak office of standards, metrology and testing. Bratislava.

[17] Goktepe AB, Lav AH (2004). Method for optimizing earthwork considering soil properties in the geometric design of highways. J. Surv. Eng..

[18] Kataguiri K, Boscov MEG, Teixeira CE, Angulo SC (2019). Characterization flowchart for assessing the potential reuse of excavation soils in Sao Paulo city. J. Clean. Prod..

[19] Lü Q, Low BK (2011). Probabilistic analysis of underground rock excavations using response surface method and SORM. Comput. Geotech..

[20] Shang J, Hencher SR, West LJ, Handley K (2017). Forensic excavation of rock masses: A technique to investigate discontinuity persistence. Rock Mech. Rock Eng..

[21] Walton G, Lato M, Anschütz H, Perras MA, Diederichs MS (2015). Non-invasive detection of fractures, fracture zones, and rock damage in a hard rock excavation—Experience from the Äspö Hard Rock Laboratory in Sweden. Eng. Geol..

[22] O'Riordan N, Phear A (2012). Measuring and mitigating the environmental impact of earthworks and other geotechnical processes. Geol. Soc. Lond. Eng. Geol. Spec. Publ..

[23] Park JY, Kim BS (2019). Life-cycle assessment-based environmental impact estimation model for earthwork-type road projects in the design phase. KSCE J. Civ. Eng..

[24] Ahmed, N., Hong, A. J., Ku, H., Moon, S., Moon, S. Technical review of automated system application to earthworks in Australia. In *ISARC. Proceedings of the International Symposium on Automation and Robotics in Construction*, Vol. 34 (IAARC Publications, 2017).

[25] Nylén EJA, Salminen JM (2019). How does the circular economy discourse affect policy-making? The case of streamlining waste utilisation in Finnish earthworks. Resour. Conserv. Recycl..

